# Huge mediastinal liposarcoma resected by clamshell thoracotomy: a case report

**DOI:** 10.1186/s40792-017-0291-5

**Published:** 2017-01-20

**Authors:** Michihito Toda, Nobuhiro Izumi, Takuma Tsukioka, Hiroaki Komatsu, Satoshi Okada, Kantaro Hara, Ryuichi Ito, Toshihiko Shibata, Noritoshi Nishiyama

**Affiliations:** Department of Thoracic Surgery, Osaka City University Medical School, 1-4-3 Asahimachi, Abeno-ku, Osaka, 545-8585 Japan

**Keywords:** Liposarcoma, Clamshell thoracotomy, Huge mediastinal tumor

## Abstract

**Background:**

Liposarcoma is the single most common soft tissue sarcoma. Because mediastinal liposarcomas often grow rapidly and frequently recur locally despite adjuvant chemotherapy and radiotherapy, they require complete excision. Therefore, the feasibility of achieving complete surgical excision must be carefully considered. We here report a case of a huge mediastinal liposarcoma resected via clamshell thoracotomy.

**Case presentation:**

A 64-year-old man presented with dyspnea on effort. Cardiomegaly had been diagnosed 6 years previously, but had been left untreated. A computed tomography scan showed a huge (36 cm diameter) anterior mediastinal tumor expanding into the pleural cavities bilaterally. The tumor comprised mostly fatty tissue but contained two solid areas. Echo-guided needle biopsies were performed and a diagnosis of an atypical lipomatous tumor was established by pathological examination of the biopsy samples. Surgical resection was performed via a clamshell incision, enabling en bloc resection of this huge tumor. Although there was no invasion of surrounding organs, the left brachiocephalic vein was resected because it was circumferentially surrounded by tumor and could not be preserved. The tumor weighed 3500 g. Pathologic examination of the resected tumor resulted in a diagnosis of a biphasic tumor comprising dedifferentiated liposarcoma and non-adipocytic sarcoma with necrotic areas. The patient remains free of recurrent tumor 20 months postoperatively.

**Conclusions:**

Clamshell incision provides an excellent surgical field and can be performed safely in patients with huge mediastinal liposarcomas.

## Background

Liposarcoma, the single most common soft tissue sarcoma, comprises 9% of primary sarcomas of the mediastinum [1]. Although the pattern of metastasis differs considerably between the histologic subtypes, mediastinal liposarcomas often grow rapidly and local recurrence (especially of myxoid and pleomorphic types) occurs frequently [2]. Because liposarcomas often grow rapidly and frequently recur locally despite adjuvant chemotherapy and radiotherapy, they require complete excision [3]. Therefore, the feasibility of achieving complete surgical excision must be carefully considered.

We here present a patient with a huge mediastinal liposarcoma that was resected by an extended surgical approach via clamshell thoracotomy.

## Case presentation

**Fig. 1:**
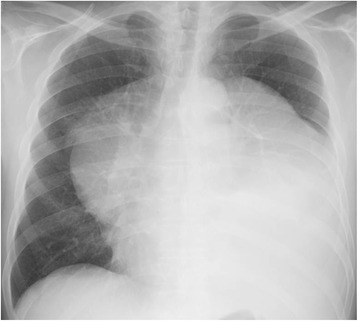
Chest X-ray film showing a huge mediastinal tumor

**Fig. 2:**
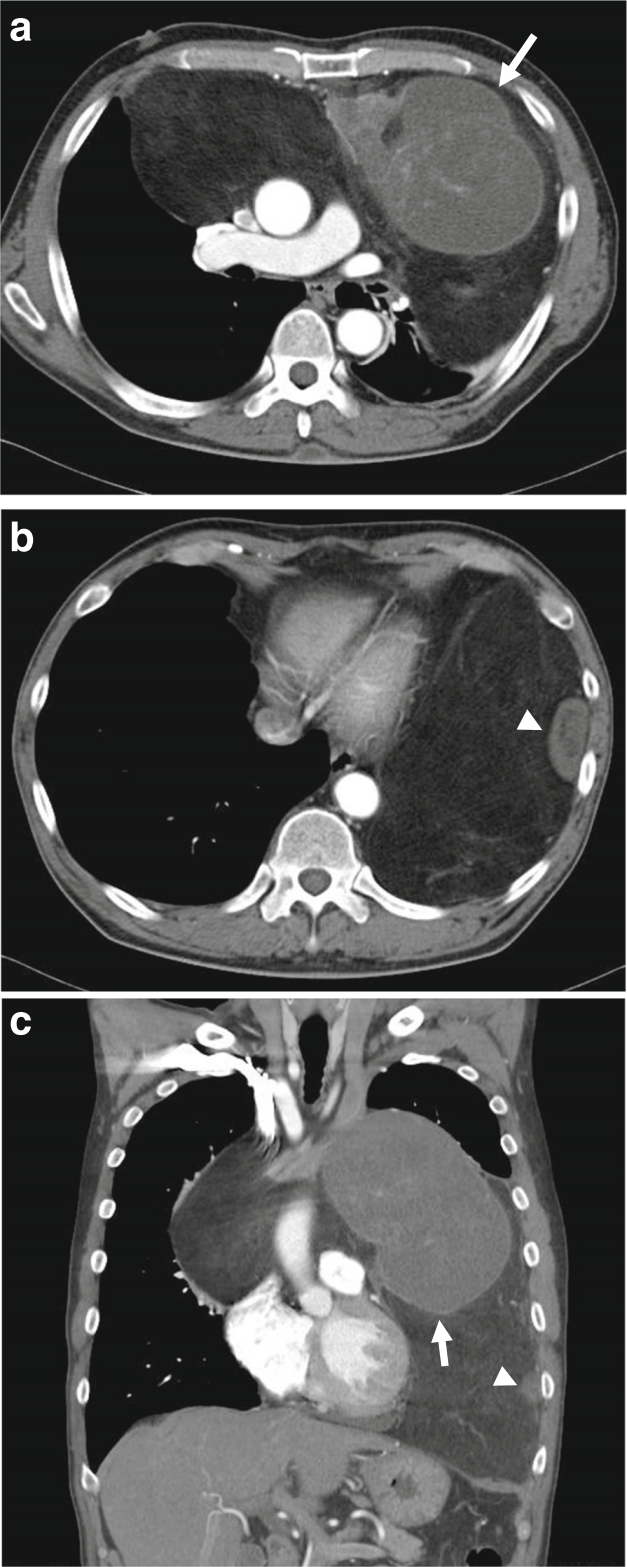
Chest CT images showing **a** a well-circumscribed mass in the anterior mediastinum measuring 36 cm × 18 cm × 10 cm and compressing the lower lobes bilaterally, **b** the tumor is composed mostly of fatty tissue but contains two solid areas, and **c** the left brachiocephalic vein is circumferentially surrounded by tumor. In these figures, the *arrow* and *arrowhead* showed each solid area

**Fig. 3:**
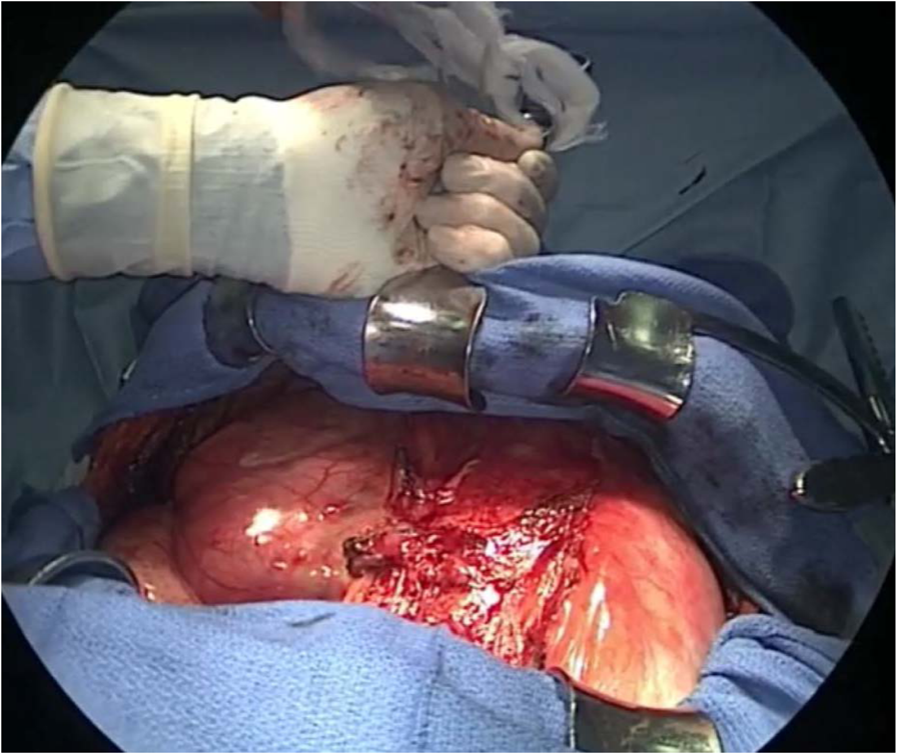
A good view and operative field was obtained by combining clamshell thoracotomy and use of multiple retractors

**Fig. 4:**
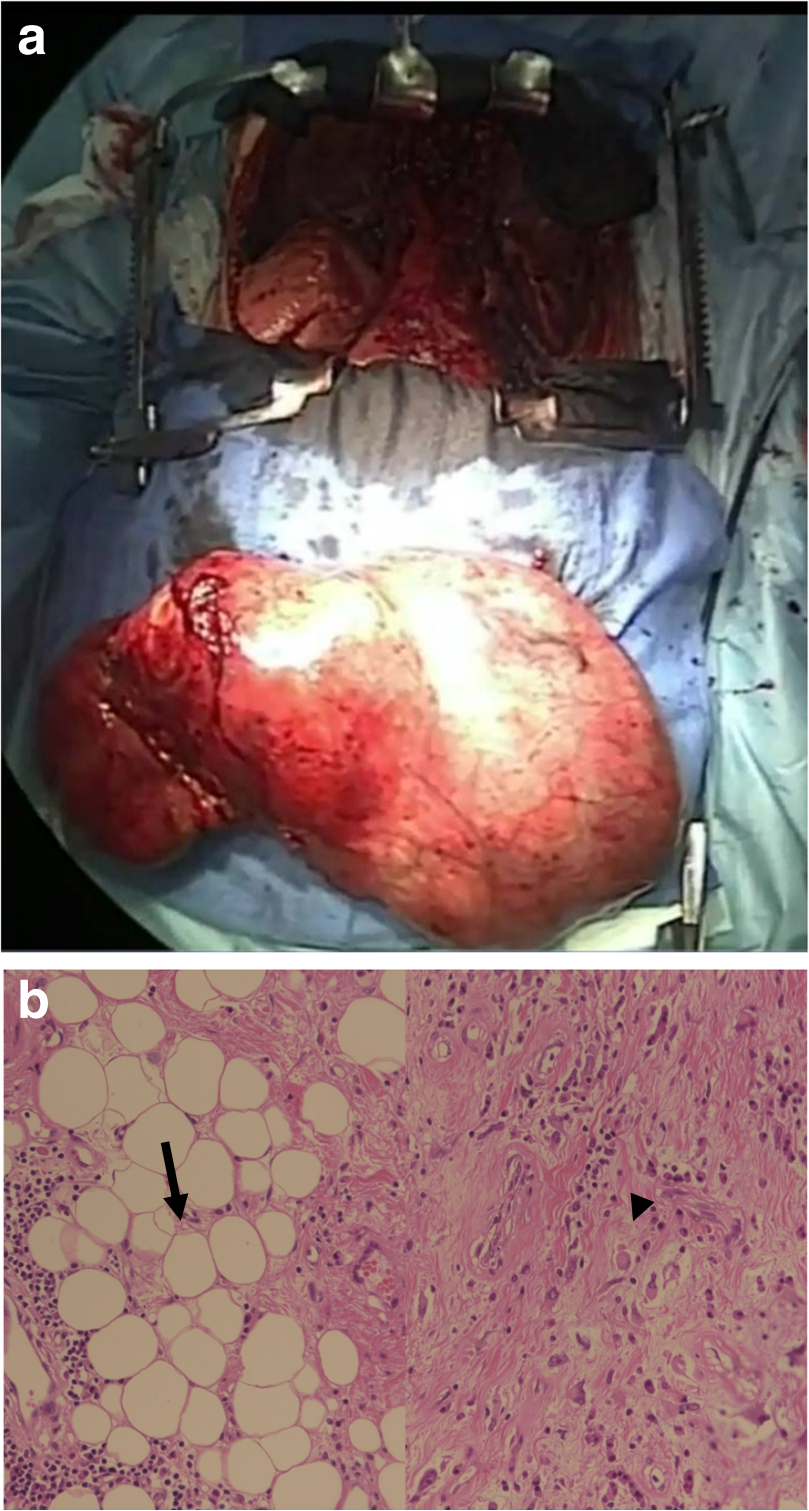
**a** The tumor weighed 3500 g and measured 36.5 cm × 18.5 cm × 10 cm. **b** The tumor was a biphasic neoplasm with a combination of a well-differentiated liposarcoma (*arrow*) and a non-adipocytic sarcoma (*arrowhead*)

A 64-year-old man presented with dyspnea on effort. Cardiomegaly had been diagnosed 6 years previously, but had been left untreated until he became symptomatic. A chest X-ray film revealed a huge mediastinal tumor (Fig. 1). Chest CT demonstrated a well-circumscribed mass in the anterior mediastinum measuring 36 cm × 18 cm × 10 cm that was compressing the lower lobe bilaterally. The tumor comprised mostly fatty tissue but contained two solid areas (Fig. 2a–c). The results of blood chemistry studies, including tumor markers, were within normal ranges. Percutaneous echo-guided needle biopsies of the lesion revealed that the fatty component was an atypical lipomatous tumor and that both solid components comprised necrotic tissue. Surgery was performed via a clamshell incision via bilateral fourth intercostal thoracotomies extending 20 cm from the midline and a transverse sternotomy. The internal thoracic arteries were ligated and divided in the process. A good field of view was achieved with the help of multiple retractors (Fig. 3). The tumor was dissected off the pleurae, pericardium, and great mediastinal vessels and mobilized from the mediastinum. As expected, the left brachiocephalic vein was circumferentially surrounded by tumor and was resected en bloc. After resection of the left brachiocephalic vein, further mobilization of the tumor was achieved. The phrenic and vagus nerves were preserved bilaterally. After completion of resection, the collapsed lungs were re-expanded by positive pressure ventilation. Because pulmonary edema did not develop after re-expansion, steroid therapy was not required. The chest was closed with two chest drains, sternal closure using steel wires, and sutures for the spread intercostal spaces. The operation time was 223 min and the total blood loss 620 mL. The tumor weighed 3500 g and measured 36.5 cm × 18.5 cm × 10 cm (Fig. 4a). Histological examination revealed a biphasic tumor comprising grade III de-differentiated liposarcoma and non-adipocytic sarcoma according to the World Health Organization (WHO) classification of soft tissue tumors (Fig. 4b) [4]. The chest drains were removed on the 13th postoperative day after ensuring that the pleural effusions were resolving. Postoperative chest X-ray films demonstrated progressive re-expansion of both lower lobes, which had been collapsed preoperatively. The patient was discharged on the 21st postoperative day. Although left phrenic nerve paralysis persisted, his dyspnea on effort improved considerably. When last seen at his 20-month follow-up visit, he reported a good quality of life and there was no evidence of recurrence.

## Discussion

Liposarcoma is the single most common soft tissue sarcoma, accounting for 20–35% of soft tissue sarcomas [2, 5]. The WHO defines four histologic liposarcoma subtypes, namely, well-differentiated, myxoid, dedifferentiated, and pleomorphic [4]. Well-differentiated liposarcomas or atypical lipomatous tumors require complete excision because of the high risk of local recurrence [6]. Two recent studies both reported that these tumors have 5-year local recurrence rates of approximately 50% [7, 8]. Because delayed dedifferentiation can occur, long-term surveillance of the operative site is recommended. En bloc resection is desirable; however, the optimal surgical technique remains controversial. Given the lack of more definitive data, we remain skeptical of the need for routine biopsies of lipomatous masses solely for the purpose of pre-operative identification of the subtype and advocate marginal excision regardless of tumor subtype. Wide local excision is also the treatment of choice for liposarcomas. In either case, an appropriate operative field is of paramount important because liposarcomas often have ill-defined borders: because they contain a high proportion of adipose-like tissue, it is sometimes difficult to distinguish tumor-related adipose tissue from normal adipose tissue [9]. Though adjuvant radiation therapy is usually reserved for higher-grade tumors in the retroperitoneum where wide surgical margins are difficult to achieve, all subtypes of liposarcoma require complete resection to minimize recurrence [10, 11].

Because primary mediastinal liposarcomas grow slowly and are relatively unlikely to invade surrounding organs, many patients remain asymptomatic until their tumors are very large. Therefore, mediastinal liposarcomas can be huge when detected. Various surgical approaches should be considered. Median sternotomy is a common approach to resecting mediastinal tumors. However, it may not afford adequate exposure of mediastinal tumors that extend into the thoracic cavity. Clamshell incisions are used to resect bilateral pulmonary metastases and large mediastinal tumors, as well as for bilateral lung transplantation. Because a component of clamshell incisions is a large transverse incision, they provide good access to the intrapleural space bilaterally. In contrast, the median vertical incision for sternotomy provides better exposure of superior and anterior mediastinal lesions. In this case, we selected a clamshell incision because we expected it to provide good visualization of the bilateral extensions of the tumor into the thoracic cavity.

As in this case, sufficiently large surgical and visual fields are essential for complete resection of huge liposarcomas. By combining clamshell thoracotomy with the use of multiple thoracotomy retractors, we were able to obtain adequate surgical and visual fields to achieve complete excision.

## Conclusions

In conclusion, we believe that clamshell incision provides an excellent surgical field and can be performed safely in patients with huge mediastinal liposarcomas.
